# Bullying Victimization and Unprotected Sex Among School-Going Adolescents in Argentina: Moderating Role of Parental Emotional Support

**DOI:** 10.1007/s40653-025-00730-0

**Published:** 2025-08-20

**Authors:** Omid Dadras

**Affiliations:** Research Centre for Child Psychiatry, University of Turku, Turun Yliopisto, Lemmkaisenkatu 3, Turku, 20520 Finland

**Keywords:** Bullying, Cyberbullying, Adolescents, Parental support, Argentina

## Abstract

Bullying, both traditional and cyber, is a pervasive issue affecting adolescents, leading to mental health issues and risky sexual behaviors. This study investigates the sex-specific relationship between bullying victimization and unprotected sex among school-going adolescents in Argentina, examining the moderating role of parental emotional support. Secondary data from the 2018 Argentina Global School-based Student Health Survey was analyzed, including 25,892 sexually active adolescents aged 12–17. Logistic regression models were used to assess the association between traditional bullying/cyberbullying and unprotected sex, stratified by sex. The moderating effect of parental emotional support was examined among bullying victims. Traditional bullying was higher among male students (27% vs. 23% in females) and was associated with higher odds of unprotected sex among males (AOR: 1.39, 95% CI: 1.10, 1.75) but not among females. Cyberbullying was higher among females (21% vs. 14% in males) and significantly increased the likelihood of unprotected sex among females (AOR: 1.41, 95% CI: 1.14, 1.74) but not among males. Parental emotional support showed a protective effect against unprotected sex for cyberbullied females (AOR: 0.75, 95% CI: 0.59, 0.96) but not for traditionally bullied adolescents of either sex. The study reveals gender-specific associations between bullying victimization and unprotected sex among Argentine adolescents. Parental emotional support appears to moderate this relationship, particularly for cyberbullied females. These findings highlight the need for gender-sensitive interventions addressing both bullying prevention and sexual health education, emphasizing the importance of parental support in mitigating risky sexual behaviors among bullied adolescents.

## Introduction

Unprotected sex among adolescents is a critical public health issue that poses significant risks, including sexually transmitted infections (STIs), unintended pregnancies, and associated psychosocial problems. The prevalence of risky sexual behaviors among young people remains alarmingly high, with studies showing that a substantial proportion of adolescents engage in unprotected sexual intercourse (CDC, 2024). Adolescents often lack comprehensive knowledge about sexual health and contraception, leading to risky behaviors that have long-term consequences on their health and well-being (Deshmukh & Chaniana, 2020). Understanding the factors that contribute to unprotected sex in this demographic is essential for developing effective interventions and educational programs (Srahbzu & Tirfeneh, 2020).

Bullying is another pervasive issue that affects adolescents, with well-documented adverse effects on mental health, academic performance, and social relationships (Le Menestrel & Rivara, 2016). It can manifest in various forms, including traditional face-to-face bullying and cyberbullying (Armitage, 2021; Waseem & Nickerson, 2017). Victims of bullying often suffer from mental health issues such as depression, anxiety, and low self-esteem (Sigurdson et al., 2015). Emerging research suggests a theoretical link between bullying experiences and sexual risk-taking behaviors, including unprotected sex. Victims may engage in risky sexual behaviors as a coping mechanism for emotional distress or due to diminished self-esteem and increased susceptibility to peer pressure (Smith et al., 2020; Tsomokos & Slavich, 2024). Adolescents who experience bullying may also develop difficulties with trust and forming healthy relationships (Tsomokos & Slavich, 2024), potentially leading to casual sexual encounters with an increased risk of unprotected sex. Additionally, victims often experience social isolation and may seek acceptance or validation through sexual relationships (Sigurdson et al., 2015), making them more susceptible to peer pressure and risky sexual activities. The anonymity and pervasive nature of cyberbullying can further exacerbate its impact, making victims feel isolated and more likely to engage in risky behaviors as a means of coping (Kim et al., 2023).

Argentina’s social landscape offers a distinctive context for examining the interplay between bullying and sexual behaviors among adolescents. The country exhibits one of the highest rates of social media penetration in Latin America, with reports indicating that over 90% of Argentine adolescents engage with platforms like Instagram and WhatsApp (Ravalli & Paoloni, 2016). This pervasive digital engagement increases exposure to cyberbullying, which may have a more severe psychological impact due to its anonymity and reach compared to traditional bullying (Sticca & Perren, 2013). Such distress could contribute to risky sexual behaviors as a coping mechanism, a pattern observed in other contexts (Hu et al., 2023). Additionally, traditional gender norms in Argentina, shaped by cultural concepts like machismo, influence how these behaviors manifest across genders(Fava et al., 2020; Rivera & Scholar, 2020). For boys, bullying victimization might prompt risky sexual activities to reaffirm masculinity (Kim et al., 2022), while girls may be more susceptible to cyberbullying targeting appearance or reputation, potentially leading to sexual risk-taking to seek validation (Dane et al., 2017; Kim et al., 2022). In particular, male adolescents may interpret traditional bullying as a challenge to their social dominance or masculine identity. Within the framework of machismo, which valorizes traits such as physical strength, emotional restraint, and sexual assertiveness, boys who are bullied may feel compelled to restore their status through externalizing behaviors like aggression, substance use, or risky sexual practices (Dunn et al., 2014; Perrotte et al., 2020). These behaviors can serve as maladaptive coping strategies, allowing male adolescents to regain a sense of control or affirm their masculinity in response to victimization (Provenzano & Boroughs, 2021). Furthermore, boys are often socialized to avoid emotional vulnerability and to resolve conflict through action rather than introspection or help-seeking, which may increase their susceptibility to engaging in high-risk behaviors following traditional bullying. These dynamics provide theoretical justification for hypothesizing a stronger link between traditional bullying and risky sexual behavior among males in this context.

Family dynamics in Argentina, particularly parental emotional support, are profoundly shaped by the country’s cultural emphasis on familial bonds, offering a potential buffer against the effects of bullying on sexual risk behaviors. Argentine culture prioritizes family as a central social institution, with parents often remaining closely involved in their children’s lives through adolescence (Herscovici, 2014). This involvement suggests that parental support could mitigate the adverse outcomes of bullying, such as unprotected sex (de Minzi et al., 2013). Recent research indicates that strong parent-adolescent relationships may serve as a protective factor against risky behaviors (Nattabi et al., 2023; Potter & Font, 2019). However, traditional gender roles may influence how this support is provided and received. Girls might benefit more from emotional support, consistent with cultural expectations of expressiveness, whereas boys may be encouraged toward independence, reducing their reliance on parental guidance (Kim et al., 2022, 2023; Nelson et al., 2017). These findings underscore the importance of exploring how Argentina’s family-centric culture and gender norms moderate the relationship between bullying and sexual risk-taking.

Despite the existing evidence linking bullying to sexual risk behaviors, significant gaps remain in understanding the underlying mechanisms and contextual factors, especially in the Argentine setting. Most studies have focused on the correlation between these behaviors, but there is a need for more research to understand the causal pathways and mediating factors. Furthermore, existing studies often have limited geographical scope and may not account for cultural variations in bullying and sexual behavior. This study aims to fill the gap by investigating how parental emotional understanding and support influence the relationship between bullying victimization (both traditional and cyberbullying) and unprotected sex among Argentine adolescents, stratified by sex. Based on the literature and the Argentine cultural context (Dadras & Takashi, 2024; Fava et al., 2020; Frezzotti, 2024; Tajer et al., 2020), we hypothesize that: (1) both traditional bullying and cyberbullying victimization are positively associated with unprotected sex; (2) the strength and direction of this association differ by gender, with traditional bullying more strongly linked to risky sexual behavior among males and cyberbullying among females; and (3) higher levels of parental emotional support mitigate the adverse effects of bullying on sexual risk-taking, particularly among female adolescents. By examining a diverse sample and considering these sex-specific moderating effects, our research aims to provide a comprehensive understanding of how bullying influences sexual risk-taking behaviors and to inform gender-specific, family-based interventions for Argentine adolescents and beyond.

## Methods

### Study Setting

This cross-sectional study is a secondary analysis of the 2018 Argentina Global School-based Student Health Survey (GSHS) dataset, a nationally representative school-based survey among students in 8th to 12th grades in Argentina. The GSHS, coordinated by the World Health Organization and supported by the Centers for Disease Control and Prevention, collects information on health behavior and protective factors among a nationally representative sample of school-going adolescents in different countries.

### Participants

Argentina’s GSHS 2018 utilized a two-stage cluster sampling method to recruit a representative sample of Argentinian students from 8th grade (primary/polymodal schools) to 12th grade (polymodal schools). In the first stage, schools were selected proportionally to the size of the school. In the second stage, classes were randomly selected, and all students in those classes were invited to participate in the survey and complete a self-administered questionnaire in Spanish. Participants were recruited within their schools during regular class sessions. No monetary or material incentives were provided, consistent with the standard protocol for the GSHS (WHO, 2025). The response rate was 86% for schools and 74% for students, with an overall response rate of 63%. A total of 56,442 adolescents aged 12–17 years old participated in the survey. Of those, 25,892 reported ever having sex and were included in this study to assess the associations of bullying victimization with unprotected sex and the moderating effect of parental understanding and monitoring.

### Study Variables

The selection of variables was guided by a comprehensive literature review on adolescent sexual risk behaviors and the availability of relevant measures in the Argentina GSHS dataset.

### Outcome Variable

*Unprotected Sex*: In GSHS, it was assessed by asking participants if they used a condom during their last sexual intercourse. The response was coded as 1 for “yes” and 0 for “no”.

### Predictor Variables

*Bullying*: Measured by asking “During the past 12 months, have you ever been bullied on school property?“: The response was binary with 1 for “yes” and 0 for “no”.

*Cyberbullying*: Measured by asking “During the past 12 months, have you ever been cyberbullied? (Count being bullied through texting, Instagram, Snapchat, Facebook, WhatsApp, Edmodo, Messenger, or other social media.)”. The response was binary with 1 for “yes” and 0 for “no”.

### Moderator Variable

*Parental understanding*: Assessed by asking “During the past 30 days, how often did your parents or guardians understand your problems and worries?“: Coded as 1 for “always/often” and 0 for “never/rarely/sometimes”.

### Covariates

Based on a comprehensive literature review, theoretical relevancy, and availability of data in GSHS, the following covariates were included in the multivariable analysis to control for potential confounding effects on the relationship between bullying victimization and unprotected sex:

*Demographic Variables*: Age (< 14, 14–15, ≥ 16), sex (male, female).

*Psychological distress*: Measured by creating a composite variable using two questions “In the past year, how frequently did you feel lonely?” and “In the past 12 months, how often were you so anxious about something that you couldn’t sleep at night?“: Coded as 1 “yes” (most of the time/always) and 0 “no” (never/rarely/sometimes).

*Substance use*: whether the participants used alcohol/marijuana/cigarette in the past 30 days: Coded 0 (no) or 1 (yes).

*Age at Sexual Debut*: <14, ≥ 14 years old.

### Statistical Analysis

Descriptive statistics were used to describe the sample demographics. The chi-square test was used to examine the differences in the distribution of the outcome (unprotected sex) and exposure variables (traditional bullying, cyberbullying) across demographic characteristics (age, grade), psychological distress, substance use, and age at sexual debut among school-going Argentinian adolescents aged 12–17 years old, stratified by sex (Table 1). Logistic regression models, adjusting for potential confounders informed by bivariate analysis, were constructed to determine the likelihood of engaging in unprotected sex among bullying victims of the opposite sex. To specify the protective effect of parental emotional support, the population was restricted to bullying victims, and the odds of unprotected sex were examined across categories of parental emotional support, with results reported as adjusted odds ratios (AOR) and 95% confidence intervals (95% CI). Missing data were treated in a listwise manner. Due to the complex sampling design in the Argentina GSHS 2018, sampling design and weights were defined and applied in all analyses using STATA 17. Statistical significance was set at *p* < 0.05.

## Results

### The Distribution of Bullying and Unprotected Sex

Table 1 illustrates the distribution and relationships between traditional bullying, cyberbullying, and unprotected sex across various demographic and psychosocial factors. A significantly higher percentage of younger adolescents (< 14 years) reported traditional bullying, with 23.2% of males and 27.4% of females being bullied. This percentage decreases with age, indicating an inverse relationship between age and traditional bullying. The trend was statistically significant (*p* < 0.001) for both males and females. The prevalence of traditional bullying varied across grades, with the highest rates in 8th grade for both males (23.0%) and females (27.1%). The prevalence decreased in higher grades, with significant differences noted for both sexes (males: *p* < 0.001, females: *p* = 0.005). Students reporting psychological distress had significantly higher rates of traditional bullying (males: 32.1%, females: 36.8%) compared to those without distress (males: 16.6%, females: 18.5%), with p-values < 0.001 for both sexes. Traditional bullying rates did not significantly differ by substance use among males (*p* = 0.866) but did among females (*p* = 0.008). A similar pattern was observed for the age at sexual debut (males: *p* = 0.653, females: *p* < 0.001).

Cyberbullying also showed significant age-related differences. Among younger adolescents, 14.1% of males and 20.9% of females reported being cyberbullied. The percentage increased with age, with the highest rates observed in adolescents aged ≥ 16 years (17.8% of males and 28.6% of females). The p-values indicate significant age differences in cyberbullying prevalence for both males (*p* = 0.013) and females (*p* < 0.001). The percentage of students experiencing cyberbullying increased with grade, particularly notable in higher grades (10th to 12th). While this increase was not statistically significant for males (*p* = 0.098), it was for females (*p* = 0.004). Similarly, cyberbullying was more prevalent among students with psychological distress (males: 30.6%, females: 41.6%) versus those without (males: 13.8%, females: 19.8%). Cyberbullying was significantly higher among substance users for both males and females. Cyberbullying did not significantly differ by age at sexual debut among males but showed a marginally significant difference among females (*p* = 0.053).

There was no significant difference in the prevalence of unprotected sex among different age groups for males, but a significant difference was observed among females (*p* = 0.031). The highest prevalence was noted among females aged ≥ 16 years (28.1%). No significant differences were observed in unprotected sex across grades for either sex. Psychological distress was associated with higher rates of unprotected sex, with significant differences observed for both males (*p* = 0.009) and females (*p* = 0.019). Substance use was associated with higher rates of unprotected sex among both males (*p* = 0.045) and females (*p* = 0.006). Age at sexual debut was significantly associated with unprotected sex for both sexes, with higher rates observed among those with an earlier sexual debut (< 14 years).

**Table 1:** The distribution of unprotected sex and bullying victimization among school-going Argentinian adolescents aged 12–17; by sex

	Total	Traditional bullying		Cyberbullying		Unprotected sex	
		Males	Females	Males	Females	Males	Females
	n (%)	(%)	(%)	(%)	(%)	(%)	(%)
Age							
< 14	10,719 (21.1)	23.2	27.4	14.1	20.9	18.8	21.9
14–15	25,758 (47.3)	18.8	25.2	16.6	27.9	17.5	23.8
≥ 16	19,965 (31.6)	16.5	20.6	17.8	28.6	18.6	28.1
*p-value*		< 0.001	< 0.001	0.013	< 0.001	0.685	0.031
Grade							
8th	8550 (14.8)	23.0	27.1	15.1	23.6	18.1	25.3
9th	11,872 (27.0)	20.0	27.3	15.1	24.5	16.5	24.7
10th	12,370 (23.3)	17.2	23.5	17.5	28.6	17.4	25.0
11th	13,137 (18.9)	18.3	22.4	17.7	29.1	19.6	28.6
12th	9474 (16.0)	16.0	20.0	17.6	27.2	19.8	25.9
*p-value*		< 0.001	0.005	0.098	0.004	0.491	0.393
Psychological distress							
No	42,841 (76.4)	16.6	18.5	13.8	19.8	17.1	24.5
Yes	13,109 (23.6)	32.1	36.8	30.6	41.6	21.5	28.5
*p-value*		< 0.001	< 0.001	< 0.001	< 0.001	0.009	0.019
Substance use							
No	52,387 (93.1)	19.0	23.9	15.7	25.5	17.6	24.8
Yes	3949 (6.9)	18.7	29.9	26.2	42.9	21.2	33.0
*p-value*		0.866	0.008	< 0.001	< 0.001	0.045	0.006
Age at sexual debut < 14							
No	13,721 (64.1)	17.2	22.6	20.0	34.3	16.1	24.8
Yes	6842 (35.9)	17.8	32.6	20.4	40.0	19.6	30.3
*p-value*		0.653	< 0.001	0.751	0.053	0.002	0.009

### Bullying Victimization on Unprotected Sex

Table 2 presents the likelihood of engaging in unprotected sex among adolescents with bullying experiences and examines the sex-specific moderating effect of parental emotional support.

Among males, adolescents who experienced bullying had a significantly higher likelihood of engaging in unprotected sex compared to those who did not experience bullying, with an AOR of 1.39 (95% CI: 1.10, 1.75). This result indicates that bullied males were 39% more likely to engage in unprotected sex. Among bullied males, those who reported receiving parental emotional support were less likely to engage in unprotected sex compared to those who did not receive such support, although this effect did not reach statistical significance.

Among females, bullying did not significantly increase the likelihood of engaging in unprotected sex, with an AOR of 1.08 (95% CI: 0.84, 1.38), indicating no statistically significant effect. Similarly, among bullied females, parental emotional support did not significantly.

**Table 2:** The likelihood of unprotected sex among adolescents with bullying experiences and sex-specific moderating effect of parental emotional support moderate the relationship between bullying and unprotected sex

	Unprotected sex	
	Males	Females
	AOR (95%CI)^1^	AOR (95%CI)^2^
Bullying		
No	Reference	Reference
Yes	1.39 (1.10, 1.75)*	1.08 (0.84,1.38)
Bullying#Parental emotional support		
Yes#No	Reference	Reference
Yes#Yes	0.64 (0.37, 1.07)	0.89 (0.53.1.50)

^1^ Adjusted for age and psychological distress. ^2^ Adjusted for age, psychological distress, substance use, and age at sexual debut < 14. * p-value < 0.05

### Cyberbullying on Unprotected Sex

Table 3 presents the likelihood of engaging in unprotected sex among adolescents with cyberbullying experiences and examines the sex-specific moderating effect of parental emotional support.

Among males, Adolescents who experienced cyberbullying had a slightly higher, but non-significant, likelihood of engaging in unprotected sex, and receiving parental emotional support was not associated with unprotected sex.

Among females, cyberbullying significantly increased the likelihood of engaging in unprotected sex, with an AOR of 1.41 (95% CI: 1.14, 1.74); indicating a 41% higher likelihood of engaging in unprotected sex. Among cyberbullied females, parental emotional support significantly moderated the relationship between cyberbullying and unprotected sex. The AOR was 0.75 (95% CI: 0.59, 0.96), indicating that cyberbullied females who received parental emotional support were 25% less likely to engage in unprotected sex compared to those who did not receive such support.

**Table 3:** The likelihood of unprotected sex among adolescents with cyberbullying experiences and the sex-specific moderating effect of parental emotional support

	Unprotected sex	
	Males	Females
	AOR (95%CI)^1^	AOR (95%CI)^2^
Cyberbullying		
No	Reference	Reference
Yes	1.24 (0.97, 1.59)	1.41 (1.14, 1.74)*
Cyberbullying#Parental emotional support		
Yes#No	Reference	Reference
Yes#Yes	0.68 (0.43,1.07)	0.75 (0.59, 0.96)*

^1^ Adjusted for age, psychological distress, and substance use. ^2^ Adjusted for age, psychological distress, substance use, and age at sexual debut < 14

## Discussion

This study examined the associations between traditional and cyberbullying victimization and the risk of unprotected sex among school-going adolescents in Argentina, with a specific focus on the moderating role of parental emotional support. Our findings indicate that traditional bullying was significantly associated with an increased likelihood of engaging in unprotected sex among male adolescents. However, this association was not significant among females. Cyberbullying showed a different pattern, where female adolescents who experienced cyberbullying were significantly more likely to engage in unprotected sex, while the association was non-significant among males. Importantly, parental emotional support moderated the relationship between cyberbullying and unprotected sex among females, providing a protective effect, but did not show a significant moderating effect in other groups. These findings underscore the importance of considering culturally specific dynamics, particularly gendered expectations and familial norms, that are inherent to the Argentine context.

Our results contribute to a growing body of literature that links bullying victimization with risky sexual behaviors. Prior studies have demonstrated that both bullies and bully-victims engage in higher rates of sexual risk behaviors, potentially as a means of coping with the emotional consequences of bullying (Holt et al., 2013). These associations may be mediated by mental health difficulties, with depressive symptoms linked to substance use, anxiety symptoms, and low self-esteem connected to sexual risk-taking (Provenzano & Boroughs, 2021). This association, however, may be influenced by gender, with girls potentially at greater risk than boys (Smith et al., 2020).

In Argentina, cultural constructs such as traditional gender roles and machismo may further intensify these gender-differentiated associations (Tajer et al., 2020). For example, the observed association between traditional bullying and unprotected sex among males may be explained by gendered cultural expectations in Argentina. As noted, traditional norms of masculinity, emphasizing dominance, emotional stoicism, and sexual assertiveness, can influence how boys respond to victimization. Risky sexual behavior may function as a maladaptive coping strategy to reassert control or masculine identity following bullying experiences (Perrotte et al., 2020; Provenzano & Boroughs, 2021). This culturally driven pressure to demonstrate toughness, along with a higher prevalence of traditional bullying among male adolescents in general, could help explain why traditional bullying is more strongly associated with unprotected sex among boys in our study. In addition, males are more prone to externalize their emotions through anger and aggressive behaviors (Dunn et al., 2014), which can lead to increased risk-taking behaviors, including risky sexual practices (Smith et al., 2020). On the other hand, boys are less likely to report cyberbullying experiences compared to girls, which may lead to an underestimation of the impact of cyberbullying on male adolescents’ sexual behaviors (Kim et al., 2023). This may explain the lack of association between cyberbullying and unprotected sex among male students in the present study.

On the other hand, the influence of cyberbullying on female adolescents appears shaped by distinct cultural processes. Argentine culture’s emphasis on social appearance and relational ties may predispose girls to internalize online harassment more deeply. Their higher engagement with social media not only increases exposure to cyberbullying but also amplifies the impact of appearance-related and relational aggression (Frezzotti, 2024). A recent study in Argentina found that digital violence against girls is prevalent, with many normalizing toxic behaviors in relationships, an acceptance that can desensitize them to violence and make risky behaviors seem necessary for social acceptance (Frezzotti, 2024; Smith et al., 2020). Victims of cyberbullying often experience heightened emotional problems, such as anxiety and depression (Man et al., 2022), which may drive them to seek validation through sexual encounters (Kim et al., 2018), or adopt risky sexual behaviors as a maladaptive coping mechanism to regain control or connection (Noll et al., 2022). This lack of effective coping can further exacerbate loneliness and lead to additional risky behaviors (Ryan, 2011). Moreover, girls are more likely to be involved in relational aggression, including social exclusion and rumor-spreading, which can undermine their social relationships and emotional well-being, potentially prompting risky sexual behaviors to maintain social connections or status (Dunn et al., 2014). Finally, as cyberbullying often targets physical appearance, and given that girls typically report lower body satisfaction and self-esteem than boys; they may be particularly vulnerable to appearance-related bullying, a factor that may contribute to the higher rates of unprotected sex observed among cyberbullying victims in our study (Provenzano & Boroughs, 2021; Smith et al., 2020).

The varying effectiveness of parental support observed in our study is consistent with research suggesting that boys and girls may respond differently to parental interventions. In Argentina, cultural values emphasizing close familial bonds and intergenerational involvement offer a unique context for understanding these differences. For instance, maternal emotional expressivity positively influences child prosocial behavior (Michalik et al., 2007), and parental warmth and emotional connection are especially salient for girls, who are socialized to value emotional support (Kincaid et al., 2012; Lippold et al., 2018). Paternal support has also been linked to reduced impulsive delinquent behavior in female juvenile offenders (Silverman & Caldwell, 2005), which may explain the lower likelihood of unprotected sex among cyberbullied females in our study. Cultural expectations in Argentina further contribute to these dynamics; while girls benefit from an environment that reinforces emotional connectedness, boys are often expected to be independent and stoic—reinforced by machismo—potentially limiting the protective effect of parental emotional support. Moreover, studies on parental meta-emotion philosophy and emotion coaching indicate that parents tend to engage more with daughters, particularly around emotions such as sadness (Morris et al., 2007). Additionally, gender differences in coping strategies, with girls favoring emotion-focused approaches like seeking social support and boys relying on problem-focused or avoidant strategies (Gundersen, 2020), further explain the differential moderating effects observed in our study. Together, these findings underscore the importance of developing gender-sensitive parenting interventions and support strategies that are informed by both cultural values and the distinct emotional needs of Argentine adolescents.

While our study primarily examines how cyberbullying contributes to risky sexual behaviors among female adolescents, it is important to acknowledge the potential for a reverse relationship. Emerging evidence suggests that adolescents who engage in risky sexual behaviors may also become targets of cyberbullying. In this context, sexual activity can sometimes be stigmatized, leading peers or online communities to judge and ostracize individuals based on their behavior (Dobson, 2019). This reverse association, whereby sexual engagement may increase vulnerability to cyberbullying, underscores the complex interplay between social norms, gender expectations, and digital interactions. However, due to the cross-sectional design of our study, we cannot establish temporal precedence or causality. Future longitudinal research should explore this bidirectional relationship to more fully understand how sexual behavior and cyberbullying mutually influence each other, which in turn could inform more comprehensive, gender-sensitive intervention strategies.

### Strengths and Limitations

One of the strengths of this study is the use of a large, nationally representative sample of Argentine adolescents, which enhances the generalizability of the findings. Additionally, the study’s focus on both traditional and cyberbullying provides a comprehensive view of the different forms of bullying victimization. However, the study has some limitations. The cross-sectional design limits our ability to establish causal relationships between bullying victimization and unprotected sex. Future research should aim to address this by employing longitudinal designs to establish causal pathways between bullying victimization and risky sexual behaviors. Additionally, self-reported data may also be subject to social desirability bias and recall bias. Another limitation of this study is that it relies on secondary data from the GSHS in which data for most variables were collected categorically. This standardized approach, while enhancing cross-national comparability and having been validated in prior research, inherently limits the precision with which associations and dose–response relationships can be examined. Additionally, the selection and inclusion of variables were restricted by what was available in the GSHS. For several aspects of adolescent health behavior that might have been of interest, the GSHS did not collect detailed information. Therefore, future research should consider primary data collection using continuous or more finely graded measures to explore these nuances further. Nonetheless, given the objectives of the GSHS and the need for internationally comparable data, the categorical format remains a robust, albeit limited, approach.

## Conclusion

Bullying, both traditional and cyber, was a significant predictor of unprotected sex among school-going adolescents in Argentina, with traditional bullying more pronounced among males and cyberbullying among females. The importance of parental emotional support in mitigating these risks suggests that strengthening family bonds and communication can play a protective role, particularly among female cyberbullying victims. Gender-specific approaches are essential, as the impact of parental support varies between males and females. By explicitly considering how Argentine cultural processes, such as machismo, familial involvement, and the high digital engagement of adolescents, influence both bullying and sexual risk behaviors, our study provides a more nuanced understanding of these interrelations. Future research should continue to explore these cultural dimensions to further refine intervention programs that resonate with the local context and effectively address the challenges faced by Argentine adolescents.

## Data Availability

The Argentina GSHS 2018 is a publicly available dataset and could be downloaded through the WHOofficial website (URL: https://extranet.who.int/ncdsmicrodata/index.php/catalog) upon a reasonable request by a registered user.
